# Outcomes of coronary artery bypass grafting (CABG) in patients with OSA-COPD overlap syndrome versus COPD alone: an analysis of US Nationwide Inpatient Sample

**DOI:** 10.1186/s12890-024-02994-y

**Published:** 2024-04-08

**Authors:** Yen-Liang Yeh, Chien-Ming Lai, Hui-Pu Liu

**Affiliations:** Division of Cardiovascular Surgery, Department of Surgery, Kaohsiung Armed Force General Hospital, No. 2, Zhongzheng 1st Rd., Lingya Dist., Kaohsiung City, Taiwan (R.O.C.)

**Keywords:** Coronary artery bypass grafting (CABG), Chronic obstructive pulmonary disease (COPD), Nationwide Inpatient Sample (NIS), Obstructive sleep apnea (OSA)

## Abstract

**Background:**

Obstructive sleep apnea (OSA) and chronic obstructive pulmonary disease (COPD) are associated with unfavorable outcomes following coronary artery bypass grafting (CABG). The purpose of this study was to compare in-hospital outcomes of patients with COPD alone versus OSA-COPD overlap after CABG.

**Methods:**

Data of adults ≥ 18 years old with COPD who received elective CABG between 2005 and 2018 were extracted from the US Nationwide Inpatient Sample (NIS). Patients were divided into two groups: with OSA-COPD overlap and COPD alone. Propensity score matching (PSM) was employed to balance the between-group characteristics. Logistic and linear regression analyses determined the associations between study variables and inpatient outcomes.

**Results:**

After PSM, data of 2,439 patients with OSA-COPD overlap and 9,756 with COPD alone were analyzed. After adjustment, OSA-COPD overlap was associated with a significantly increased risk of overall postoperative complications (adjusted odd ratio [aOR] = 1.12, 95% confidence interval [CI]: 95% CI: 1.01–1.24), respiratory failure/prolonged mechanical ventilation (aOR = 1.27, 95%CI: 1.14–1.41), and non-routine discharge (aOR = 1.16, 95%CI: 1.03–1.29), and AKI (aOR = 1.14, 95% CI: 1.00-1.29). Patients with OSA-COPD overlap had a lower risk of in-hospital mortality (adjusted odd ratio [aOR] = 0.53, 95% CI: 0.35–0.81) than those with COPD only. Pneumonia or postoperative atrial fibrillation (AF) risks were not significantly different between the 2 groups. Stratified analyses revealed that, compared to COPD alone, OSA-COPD overlap was associated with increased respiratory failure/prolonged mechanical ventilation risks among patients ≥ 60 years, and both obese and non-obese subgroups. In addition, OSA-COPD overlap was associated with increased risk of AKI among the older and obese subgroups.

**Conclusion:**

In US adults who undergo CABG, compared to COPD alone, those with OSA-COPD are at higher risks of non-routine discharge, AKI, and respiratory failure/prolonged mechanical ventilation, but a lower in-hospital mortality. No increased risk of AF was noted.

**Supplementary Information:**

The online version contains supplementary material available at 10.1186/s12890-024-02994-y.

## Background

Coronary artery bypass grafting (CABG) is aimed at restoring blood flow to the heart by bypassing blocked or narrowed coronary arteries. More than 200,000 patients undergo CABG in the United States (US) annually [1]. Approximately 14% of patients go to the emergency department (ED) within 30 days after CABG, for a range of postoperative complications including graft malfunction, sternal wound infection, pneumonia, stroke, venous thromboembolic events (VTEs), atrial fibrillation (AF), pulmonary hypertension, pericardial effusion, kidney function impairment, gastrointestinal issues, and hemodynamic instability [2, 3]. The risk factors contributing to perioperative mortality and morbidity associated with CABG can be categorized into 3 main groups: patient-related attributes, characteristics of the healthcare providers, and factors arising after the surgical procedure itself [3]. During the CABG procedure, a patient is placed on cardiopulmonary bypass in which a machine replaces the functions of the heart and lungs [4]. Once the procedure is completed, bypass is discontinued and heart and lung functions are restored.

A target oxygen saturation of 94 to 98% in most ill patients is recommended by the British Thoracic Society guideline [5]. Lower oxygen saturations are associated with an increased risk of death from pulmonary diseases [6]. Among the diseases that compromise respiratory function, chronic obstructive pulmonary disease (COPD) and obstructive sleep apnea (OSA) are known to affect blood oxygen level [7, 8], and both conditions are established risk factors for premature death [9, 10]. Notably, COPD and OSA frequently co-exist or overlap, and this convergence is referred to as OSA-COPD overlap. The overlap of the 2 conditions has an established clinical relevance, but lacks proper recognition within the broader community of respiratory health experts. Individuals affected by the overlap between OSA and COPD often have increased respiratory symptoms and a diminished quality of life. Moreover, the likelihood of experiencing exacerbations, hospital admissions, and mortality exceeds what is typically linked to each individual condition on its own [11, 12].

Previous studies have linked OSA to adverse outcomes in patients undergoing CABG, including a higher risk of postoperative complications such as respiratory distress, arrhythmias, and prolonged hospital stays [13, 14]. Similarly, individuals with COPD who undergo CABG also have increased risks of perioperative complications, including respiratory infections, longer mechanical ventilation, and prolonged hospitalization [15, 16]. Recently, a study by Desai et al. [17] examined the influence of OSA-COPD on outcomes following percutaneous coronary intervention (PCI), and demonstrated that patients with OSA-COPD overlap experienced worse outcomes than those with OSA alone. However, no study has yet evaluated the influence of OSA-COPD overlap in the setting of CABG.

Given the substantial gap within the medical literature and the far-reaching consequences that complications following CABG procedures pose on the healthcare system [18], we conducted this comprehensive study utilizing a nationwide dataset. The aim of our investigation was to thoroughly compare the in-hospital outcomes subsequent to CABG, specifically focusing on patients with sole COPD diagnoses versus those with OSA-COPD.

## Methods

### Data source

This population-based, retrospective observational study used data extracted from the US Nationwide Inpatient Sample (NIS) database, which is the largest all-payer, continuous inpatient care database in the US, and includes information of about 8 million hospital stays each year [19]. The database is administered by the Healthcare Cost and Utilization Project (HCUP) (http://ahrq.gov/data/hcup/index.html) of the US National Institutes of Health (NIH). Patient data include primary and secondary diagnoses, primary and secondary procedures, admission and discharge status, patient demographics, expected payment source, duration of hospital stay, and hospital characteristics (i.e., bed number/location/teaching status/hospital region). All admitted patients are initially considered for inclusion. The continuous, annually updated NIS database derives patient data from about 1,050 hospitals from 44 states in the US, representing a 20% stratified sample of US community hospitals as defined by the American Hospital Association.

### Ethics statement

This study complies with the terms of the NIS data-use agreement. Given that this study solely involved the analysis of secondary data, there was no direct involvement of the general public or patients. It was granted exemption from requiring IRB approval.

### Study design and patients

The data of adult patients ≥ 18 years old admitted to US hospitals between 2005 and 2018 with a diagnosis of COPD who received CABG were extracted from the NIS database. Patients without information of sex, race, and the main study outcomes were excluded. Patients who had asthma, received concurrent heart valve surgery, or an emergent surgery were also excluded. Patients receiving emergent CABG were excluded to enhance the uniformity of the study population. These diagnoses and procedures were identified using the International Classification of Diseases, Ninth and Tenth edition (ICD-9 and ICD-10) codes, as summarized in Supplementary Table S1.

Patients included were separated into 2 groups; those with OSA and those without OSA.

### Outcome measures

The outcomes were in-hospital mortality, non-routine discharge (defined as discharge to long-term care facilities), length of hospital stays (LOS), total hospital costs, and the occurrence of any or specific postoperative complications. The complications assessed were bleeding, postoperative shock, venous VTE, pneumonia, infection/sepsis, respiratory failure/prolonged mechanical ventilation, acute kidney injury (AKI), and postoperative atrial fibrillation (AF). Prolonged mechanical ventilation was defined as mechanical ventilation usage over 96 consecutive hours. These complications were identified through the ICD codes documented in Supplementary Table S1.

### Covariates

Patient demographic information extracted included age, sex, race, insurance status, household income, smoking, and year of surgery. Clinical characteristics included major comorbidities that were identified using the ICD code system. Comorbidities included in this analysis were obesity (defined as a body mass index [BMI] ≥ 30 kg/m^2^), congestive heart failure, valvular heart disease, chronic kidney disease (CKD), peripheral vascular disease, obesity, history of myocardial infarction (MI), prior percutaneous coronary intervention (PCI), continuous positive airway pressure (CPAP) use, previous CABG or heart valve surgery, history of AF, diabetes, and Charlson Comorbidity Index (CCI). Hospital-related characteristics such as bed number and location/teaching status were extracted from the database as part of the comprehensive data available for all participants.

### Statistical analysis

Since the NIS database covers a 20% sample of the US annual inpatient admissions, weighted samples (before 2011 using TRENDWT & after 2012 using DISCWT), stratum (NIS_STRATUM), cluster (HOSPID) were used to produce national estimates for all analyses. SAS software provides analysis of sample survey data using the SURVEY procedure. Descriptive statistics of the patients with COPD who received CABG were presented as number (n) and weighted percentage (%), or mean and standard error (SE). Categorical data were analyzed using PROC SURVEYFREQ, and continuous data were analyzed using PROC SURVEYREG. To balance the baseline characteristics between the comparison groups (i.e., OSA-COPD overlap vs. COPD alone), propensity score matching (PSM) according to sex, study year, obesity status, and age-adjusted CCI (ACCI) was used to achieve a case: control ratio of 1:4. The ACCI modifies the CCI by incorporating age, adding one point for each decade beyond 40 years, with a maximum addition of four points. The odds ratio (OR) and 95% confidence interval (CI) were calculated for the associations between the outcomes and study variables using the logistic regression analysis. The estimated value and 95% CI were calculated using the linear regression analysis. The covariates with significant differences between the 2 comparison groups after PSM were identified as the adjusted variables in multivariable regression. All p values were 2-sided, and a value of *p* < 0.05 was considered statistically significant. All statistical analyses were performed using the SAS software version 9.4 (SAS Institute Inc., Cary, NC, US).

## Results

### Study population

**Fig. 1:**
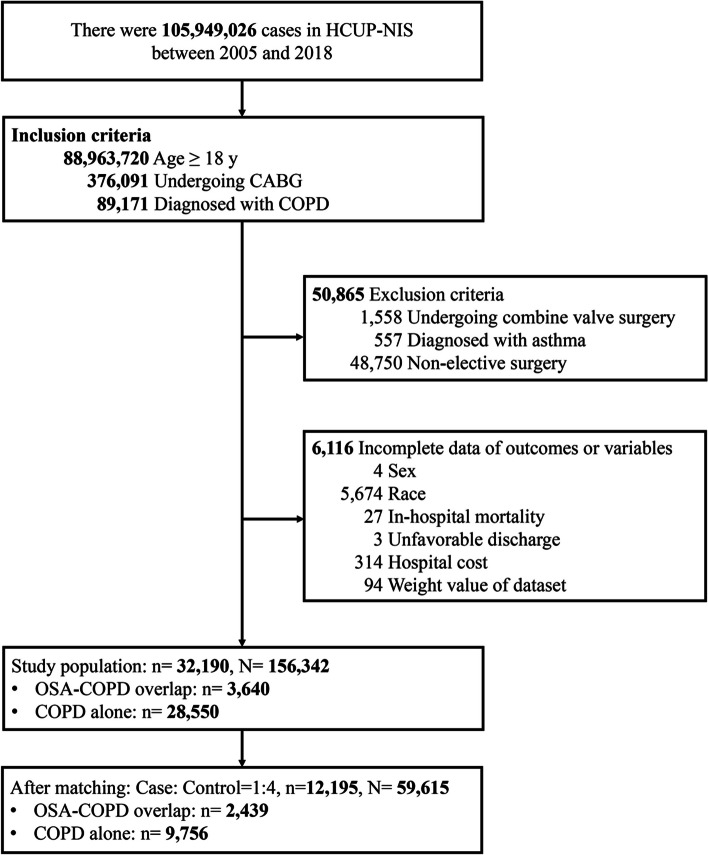
Flow diagram of patient selection process

A flow diagram of patient selection is shown in Fig. 1. A total of 89,171 patients ≥ 18 years old with COPD who underwent CABG between 2005 and 2018 were identified in the database. After excluding patients with asthma, undergoing valve surgeries, or an emergent surgery (*n* = 50,865), or had missing information on study outcomes or variables (*n* = 6,116), 32,190 were included as the study population. After PSM, 12,195 patients were left for subsequent analyses. This patient sample could be extrapolated to a total of 59,615 adults in the US after applying the sample weights provided by the database. Of the patients, 2,439 (20.0%) patients had OSA-COPD overlap, and 9,756 (80.0%) had COPD alone (Fig. 1).

### Characteristics of the study population after PSM

Patient outcomes, demographic data comorbidities, and hospital status after PSM are summarized in Table 1. The mean age of the patients was 65.8 ± 0.1 years, 9,273 (75.9%) patients were males, and 10,531 (86.4%) were White. There were still significant differences of race distributions, insurance status, household income, smoking, hospital location/teaching status, CKD, peripheral vascular disease, history of AF, diabetes, CPAP, prior PCI, and ACCI between patients with OSA-COPD overlap and patients with COPD alone (all, *p* < 0.05).

**Table 1:** Characteristics and in-hospital outcomes of the study population after PSM

Characteristics	Total (*N* = 12,195)	OSA-COPD overlap (*n* = 2,439)	COPD alone (*n* = 9,756)	*p*
**In-hospital mortality**	191 (1.6)	24 (1.0)	167 (1.7)	**0.006**
**LOS, days**^**a**^	8.3 ± 0.1	8.3 ± 0.1	8.4 ± 0.1	0.497
**Non-routine discharge**^**a**^	2457 (20.6)	558 (23.2)	1899 (19.9)	**< 0.001**
**Total hospital costs, per 1000 dollars**	148.3 ± 1.5	145.8 ± 2.2	149.0 ± 1.6	0.208
**Postoperative complications**	8353 (68.6)	1741 (71.4)	6612 (67.9)	**< 0.001**
Bleeding	6159 (50.7)	1214 (50.0)	4945 (50.9)	0.457
Postoperative shock	597 (4.9)	110 (4.6)	487 (5.0)	0.380
VTE	218 (1.8)	43 (1.7)	175 (1.8)	0.874
Pneumonia	710 (5.8)	147 (6.0)	563 (5.8)	0.647
Infection/sepsis	735 (6.0)	143 (5.8)	592 (6.0)	0.671
Respiratory failure / prolonged mechanical ventilation	2618 (21.4)	631 (25.8)	1987 (20.3)	**< 0.001**
AKI	1730 (14.3)	397 (16.4)	1333 (13.7)	**< 0.001**
Postoperative AF	1284 (10.5)	271 (11.0)	1013 (10.4)	0.333
**Age, years**	65.8 ± 0.1	65.6 ± 0.2	65.9 ± 0.1	0.218
18–49	566 (4.6)	101 (4.1)	465 (4.7)	0.169
50–59	2451 (20.1)	527 (21.6)	1924 (19.7)	
60–69	4809 (39.4)	965 (39.5)	3844 (39.4)	
70–79	3614 (29.7)	704 (28.9)	2910 (29.9)	
80+	755 (6.2)	142 (5.9)	613 (6.3)	
**Sex**				0.253
Male	9273 (75.9)	1834 (75.1)	7439 (76.2)	
Female	2922 (24.1)	605 (24.9)	2317 (23.8)	
**Race**				**< 0.001**
White	10,531 (86.4)	2164 (88.7)	8367 (85.8)	
Black	637 (5.2)	127 (5.2)	510 (5.3)	
Hispanic	568 (4.7)	86 (3.5)	482 (4.9)	
Others	459 (3.7)	62 (2.5)	397 (4.0)	
**Insurance status**				**< 0.001**
Medicare/Medicaid	8183 (67.3)	1621 (66.6)	6562 (67.5)	
Private including HMO	3361 (27.5)	719 (29.5)	2642 (27.0)	
Self-pay/no-charge/other	639 (5.2)	96 (3.9)	543 (5.5)	
Missing	12	9		
**Household income**				**< 0.001**
Quartile1	3955 (33.2)	719 (30.1)	3236 (33.9)	
Quartile2	3586 (30.0)	675 (28.3)	2911 (30.5)	
Quartile3	2712 (22.7)	588 (24.6)	2124 (22.2)	
Quartile4	1683 (14.1)	405 (16.9)	1278 (13.4)	
Missing	259	52	207	
**Smoking**				**< 0.001**
No	3840 (31.3)	863 (35.1)	2977 (30.3)	
Yes	8355 (68.7)	1576 (64.9)	6779 (69.7)	
**Study year**				0.777
2005–2009	3515 (27.8)	685 (27.3)	2830 (28.0)	
2010–2015	4818 (39.8)	979 (40.3)	3839 (39.6)	
2016–2018	3862 (32.4)	775 (32.4)	3087 (32.4)	
**Hospital bed number**				0.072
Small	1163 (9.3)	229 (9.2)	934 (9.4)	
Medium	2997 (24.8)	645 (26.7)	2352 (24.4)	
Large	7978 (65.9)	1548 (64.1)	6430 (66.3)	
Missing	57	17	40	
**Hospital location/ teaching status**				**0.004**
Rural	573 (4.7)	85 (3.5)	488 (5.0)	
Urban nonteaching	4123 (33.6)	814 (33.5)	3309 (33.6)	
Urban teaching	7442 (61.7)	1523 (63.1)	5919 (61.4)	
Missing	57	17	40	
**Comorbidity**				
Congestive heart failure	3480 (28.7)	681 (28.1)	2799 (28.8)	0.498
Valvular heart disease	1083 (8.9)	214 (8.8)	869 (8.9)	0.799
CKD	1997 (16.5)	471 (19.4)	1526 (15.8)	**< 0.001**
Peripheral vascular disease	3128 (25.7)	568 (23.3)	2560 (26.3)	**0.003**
Obesity	3685 (30.0)	737 (30.1)	2948 (30.0)	0.970
History of MI	2619 (21.6)	497 (20.5)	2122 (21.9)	0.143
History of AF	4005 (33.0)	886 (36.5)	3119 (32.1)	**< 0.001**
Diabetes	5372 (44.1)	1277 (52.4)	4095 (42.0)	**< 0.001**
CPAP use	608 (5.0)	235 (9.7)	373 (3.8)	**< 0.001**
Prior PCI	1307 (10.7)	295 (12.1)	1012 (10.3)	**0.012**
Prior valvular surgery	37 (0.3)	11 (0.4)	26 (0.3)	0.173
Prior CABG	238 (2.0)	46 (1.9)	192 (2.0)	0.789
**ACCI**				0.534
0	115 (0.9)	22 (0.9)	93 (0.9)	
723 (5.9)	140 (5.7)	583 (5.9)		
1707 (13.9)	352 (14.3)	1355 (13.8)		
3	2626 (21.5)	496 (20.3)	2130 (21.8)	
4	2446 (20.0)	484 (19.8)	1962 (20.1)	
5+	4578 (37.7)	945 (39.0)	3633 (37.4)	

*Abbreviations*: *LOS* Length of hospital stays, *VTE* Venous thromboembolism, *AKI* Acute kidney injury, *AF* Atrial fibrillation, *HMO* Health Maintenance Organization, *CKD* Chronic kidney disease, *MI* Myocardial infarction, *CPAP* Continuous positive airway pressure, *PCI* Percutaneous coronary intervention, *CABG* Coronary artery bypass grafting, *ACCI* Age-adjusted Charlson Comorbidity Index

Continuous variables are presented as mean ± SE; categorical variables are presented as unweighted counts (weighted percentage)

^a^Excluding patients who died in the hospital

*p*-values < 0.05 are shown in bold

With respect to in-hospital outcomes, the frequencies of in-hospital mortality, non-routine discharge, postoperative complications (any), respiratory failure/mechanical ventilation, and AKI differed significantly between the 2 groups (all, *p* < 0.05) (Table 1).

Characteristics of patients before PSM are summarized in Supplementary Table S2.

### Associations between OSA-COPD overlap versus COPD alone, postoperative complications, and non-routine discharge following CABG

The association between OSA-COPD overlap versus COPD alone and outcomes are summarized in Table 2. After adjustment, patients with OSA-COPD overlap had a significantly increased risk of non-routine discharge (aOR = 1.16, 95% CI: 1.03, 1.29), postoperative complications (aOR = 1.12, 95% CI: 1.01, 1.24), respiratory failure/prolonged mechanical ventilation (aOR = 1.27, 95% CI: 1.14, 1.41), and AKI (aOR = 1.14, 95% CI: 1.00, 1.29). Conversely, patients with OSA-COPD overlap had a lower risk of in-hospital mortality (aOR = 0.53, 95% CI: 0.35, 0.81) than those with COPD only. There was no significant difference in LOS (adjusted Beta (aBeta): -0.19, 95% CI: -0.42, 0.04). Similarly, no significantly increased risks of pneumonia (aOR = 0.96, 95% CI: 0.79, 1.16), or postoperative AF (aOR = 1.04, 95% CI: 0.9, 1.20) were observed between patients with OSA-COPD overlap and COPD alone (Table 2). The complete model of the associations between study variables and outcomes is shown in Supplementary Table S3.

**Table 2:** Associations between OSA-COPD overlap versus COPD alone, and in-hospital outcomes

Outcomes	Univariate		Multivariable	
	OR/ Beta (95% CI)	*p*	aOR/ aBeta (95% CI)	*p*
**In-hospital mortality**^a^	0.56 (0.37, 0.85)	**0.007**	0.53 (0.35, 0.81)	**0.003**
**LOS**^b, e^	-0.03 (-0.25, 0.19)	0.809	-0.19 (-0.42, 0.04)	0.098
**Non-routine discharge**^c, e^	1.21 (1.09, 1.35)	**< 0.001**	1.16 (1.03, 1.29)	**0.012**
**Postoperative complications, any**^d^	1.18 (1.07, 1.31)	**< 0.001**	1.12 (1.01, 1.24)	**0.028**
Pneumonia^d^	1.04 (0.87, 1.25)	0.647	0.96 (0.79, 1.16)	0.669
Respiratory failure / prolonged mechanical ventilation^d^	1.37 (1.24, 1.51)	**< 0.001**	1.27 (1.14, 1.41)	**< 0.001**
AKI^d^	1.23 (1.09, 1.39)	**< 0.001**	1.14 (1.00, 1.29)	**0.044**
Postoperative AF^d^	1.07 (0.93, 1.23)	0.333	1.04 (0.90, 1.20)	0.608

*Abbreviations*: *LOS* Length of hospital stays, *AKI* Acute kidney injury, *AF* Atrial fibrillation, *CPAP* Continuous positive airway pressure, *PCI* Percutaneous coronary intervention, *OR* Odds ratio, *aOR* adjusted odds ratio, *aBeta* adjusted odds ratio, *CI* Confidence interval

*p*-values < 0.05 are shown in bold

^a^Adjusted for insurance status, smoking, hospital bed number, prior PCI and prior valvular surgery

^b^Adjusted for sex, race, household income, insurance status, smoking, hospital bed number, hospital location/ teaching status, CPAP use, prior valvular surgery and prior PCI

^c^Adjusted for sex, household income, insurance status, smoking, CPAP use and prior PCI

^d^Adjusted for sex, race, household income, insurance status, smoking, hospital location/ teaching status, CPAP use and prior PCI

^e^Excluding patients who died in the hospital

### Associations between OSA-COPD overlap versus COPD alone and in-hospital outcomes following CABG, stratified by age and obesity status

Stratified associations between OSA-COPD overlap versus COPD alone and outcomes are summarized in Table 3. Patients with OSA-COPD overlap had a significantly increased risk of respiratory failure/prolonged mechanical ventilation (aOR = 1.27, 95% CI: 1.12, 1.43) compared to patients with COPD alone among the subgroup of patients older than 60 years but not their younger counterparts.

**Table 3:** Associations between OSA-COPD overlap versus COPD alone, respiratory failure / prolonged mechanical ventilation, postoperative AF, and stratified AKI, by age and obesity status

Subgroup	Respiratory failure / prolonged mechanical ventilation		AKI		Postoperative AF	
	aOR (95% CI)	*p*	aOR (95% CI)	*p*	aOR (95% CI)	*p*
**Age < 60 years**	1.21 (0.99, 1.49)	0.062	1.02 (0.75, 1.40)	0.889	1.21 (0.90, 1.64)	0.200
**Age ≥ 60 years**	1.27 (1.12, 1.43)	**< 0.001**	1.20 (1.04, 1.37)	**0.009**	0.99 (0.85, 1.17)	0.936
**Non-obese**	1.20 (1.06, 1.36)	**0.004**	1.10 (0.95, 1.28)	0.200	0.99 (0.83, 1.18)	0.931
**Obese**	1.40 (1.16, 1.69)	**< 0.001**	1.31 (1.05, 1.63)	**0.016**	1.07 (0.85, 1.37)	0.559

Adjusted for sex, race, household income, insurance status, smoking, hospital location/ teaching status, CPAP use and prior PCI

*Abbreviations*: *AKI* Acute kidney injury, *CPAP* Continuous positive airway pressure, *PCI* Percutaneous coronary intervention, *AF* Atrial fibrillation, *OR* Odds ratio, *aOR* adjusted odds ratio, *CI* Confidence interval

In addition, OSA-COPD overlap was associated with increased risk of respiratory failure/prolonged mechanical ventilation compared to COPD alone regardless of obesity status (non-obese: aOR = 1.20, 95% CI: 1.06, 1.36; obese: aOR = 1.40, 95% CI: 1.16, 1.69).

Further, OSA-COPD overlap was significantly associated with increased risk of AKI among both the older and obese subgroups (age ≥ 60 years: aOR = 1.20, 95% CI: 1.04, 1.37; obese: aOR = 1.31, 95% CI: 1.05, 1.63).

OSA-COPD was not significantly associated with an increased risk of postoperative AF (*p* > 0.05) in all subgroups, as compared to COPD alone (Table 3).

## Discussion

To the best of our knowledge, this study is the first to investigate outcomes following elective CABG between patients with OSA-COPD overlap versus COPD alone. The analyses found that of patients with COPD and undergoing CABG, 20% had OSA-COPD overlap syndrome. Compared to patients with COPD alone, those with OSA-COPD overlap are more likely to have a non-routine discharge, and overall postoperative complications. Additionally, compared to patients with COPD alone, those with OSA-COPD overlap have a higher likelihood of respiratory failure/prolonged mechanical ventilation, regardless of obesity status. Furthermore, OSA-COPD is associated with a higher risk of AKI among obese patients or patients older than 60 years. Nevertheless, OSA-COPD appears to not be associated with a greater risk of postoperative AF or pneumonia than COPD alone. Unexpectedly, in-hospital mortality risk appears lower in patients with OSA-COPD overlap than COPD alone after CABG.

While PCI is a commonly performed procedure, CABG has been performed for more than 50 years and is preferred over PCI in patients with very severe atherosclerosis [4]. Patients who require CABG commonly have comorbidities that can increase the risk of surgery, and 2 comorbidities commonly seen in patients who require CABG are COPD and OSA [4]. Both COPD and OSA are associated with a decreased oxygen saturation, and in OSA-COPD overlap the decrease can be greater than in either condition alone, resulting in increased morbidity and mortality [11, 17, 20].

COPD is a chronic lung disease associated with decreased oxygenation that can be progressive, and debilitating [7, 9, 20]. Patients with COPD who require cardiac surgery, including CABG, are at increased risk of complications. A study comparing the outcomes of cardiac surgery in patients with COPD and those without COPD reported that patients with COPD required a longer intubation time, longer ICU stay, and longer LOS in the hospital [16]. Patients with COPD also had a higher risk of postoperative bronchoconstriction, respiratory failure, and AF. The mortality rate within 30 days after surgery was also higher in patients with COPD than those without. A meta-analysis of patients with COPD undergoing CABG found that COPD was associated with higher risks of postoperative pneumonia, respiratory failure, stroke, renal failure, and wound infection [15].

Like COPD, OSA is a chronic condition that is characterized by complete or partial obstruction of airflow during sleep [10, 20]. Typical symptoms of OSA are daytime sleepiness and fatigue; however, persons with OSA are at increased risk of heart failure, arrhythmias, and coronary artery disease [10]. While it is difficult to estimate the overall prevalence of OSA-COPD overlap syndrome, it is believed than more than 10% of adults have COPD and 9 to 38% of adults have OSA, and OSA is typically associated with male sex, obesity, and advanced age [12]. In addition, it is believed that COPD can increase the risk of developing OSA, and OSA increases the risk of acute exacerbations of COPD [12]. Patients with OSA-COPD overlap have more severe respiratory symptoms, worse quality of life, a higher rate of hospitalizations, and higher mortality than persons with either condition alone [12, 21]. Additionally, patients with OSA-COPD overlap have a higher prevalence of hypertension and diabetes than those with COPD alone [21, 22]. Risk factors for OSA in patients with COPD include high BMI, neck circumference, and CCI score [21]. Interestingly, the risk of OSA was found to be lower in patients with severe COPD than those with mild or moderate COPD [21]. Recent studies have reported OSA-COPD overlap syndrome is associated with increased cardiovascular risk, including hypertension, pulmonary hypertension, heart failure, ischemic heart disease, and cerebrovascular disease [23–25].

In the present study, we found that patients with OSA-COPD overlap had increased risks of various inpatient outcomes, which is generally consistent with the previous literature. However, we unexpectedly found that patients with OSA-COPD overlap had a lower risk of in-hospital mortality than those with COPD alone. While seemingly paradoxical, a number of studies in the literature have documented similar phenomena. Raymonde et al. also reported that in patients with pneumonia who were on mechanical ventilation, OSA was linked to increased non-routine discharges but lower in-hospital mortality [26]. Another study highlighted that while OSA leads to a higher comorbidity burden and slightly increased complication rates in patients undergoing spinal fusions, it does not independently predict inpatient mortality [27]. Yet another research using the same NIS dataset suggested that OSA is associated with a reduced in-hospital mortality among non-surgical patients [28]. The authors stated that the unexpected result may arise from the failure to identify patients with undiagnosed OSA. Other potential explanations may be: Patients with OSA may receive more comprehensive respiratory management, including the use of CPAP, thereby lessening the hypoxemia often seen in COPD. This might also, in turn, reduce pulmonary hypertension, and decrease the workload on the heart, and mitigate some of the adverse effects of COPD on the cardiovascular system, potentially reducing the risk of life-threatening events. Also, patients diagnosed with both OSA and COPD might undergo more rigorous and frequent medical surveillance than those with COPD alone. This can lead to earlier detection and treatment of potential complications, thereby reducing the risk of mortality. It’s important to note that while these factors might help explain the observed protective effect of OSA-COPD overlap on in-hospital mortality, it should be subject to ongoing verification through future research. Lastly, although already considered in our analysis, the use of CPAP might be largely underestimated when relying on admission claim codes, leading to a potential bias.

Notably, our findings indicate that both hospital bed number and location significantly impact in-hospital outcomes, with smaller bed numbers associated with higher mortality and rural hospitals showing lower risk or respiratory failure compared to urban-teaching hospitals, as detailed in Supplementary Table S3. Hospitals with fewer beds often have lower patient volumes, potentially influence the surgical experience and quality of care. Urban and teaching hospitals, dealing with complex cases and specializing in certain treatments, might show higher respiratory failure rates due to the nature of their patient demographics and the complexity of cases they handle.

Despite expectations of higher costs due to the poorer outcomes associated with OSA-COPD overlap syndrome, our study discovered that total hospital costs did not significantly differ from those for COPD alone. This can be attributed to the overlap in treatment pathways and healthcare resource utilization for both conditions, including the use of non-invasive ventilation techniques common to their management. Consequently, the presence of OSA-COPD overlap does not substantially increase healthcare costs beyond the treatment expenses for COPD alone.

Though our results did not find any excessive risks of OSA-COPD on the occurrence of AF compared to COPD alone, other studies have reported relations between COPD, OSA, and heart surgery. AF is the most common atrial arrhythmia after CABG, with a prevalence of 15 to 45%, and is associated with a poor long-term prognosis [29]. A study of postoperative AF in patients with OSA who underwent CABG reported that all categories of OSA were significantly associated with postoperative AF, with the greatest association for severe OSA (OR = 6.82) [30]. It is estimated that about 24% of patients with AF also have COPD [31]. It is also believed that COPD promotes the progression of AF, increases the recurrence of AF after cardioversion, and reduces the effectiveness of AF treatment [31]. A recent meta-analysis reported that about 13% of patients with AF also have COPD, and the presence of COPD is associated with overall worse outcomes and a 2-fold increased risk of all-cause death, cardiovascular death, and major bleeding [32].

### Strengths and limitations

This strength of this study stems from its use of a large, comprehensive sample that is representative of the entire US, which allows for proper generalization of the findings. The groups in the study were intricately matched and finely adjusted, demonstrating a meticulous approach to mitigate potential confounding effects on the measured variables. However, it’s important to acknowledge that the inherent limitations of the study come from its retrospective and observational design. These constraints could potentially hinder the accurate measurement of specific variables, and the possibility of selection bias cannot be entirely eliminated. Similar to other studies that utilize ICD coding systems, it’s worth noting that the potential for coding errors cannot be entirely dismissed in this study as well. Of utmost significance, it’s crucial to recognize that the administrative codes used in this study do not allow for the differentiation of COPD and OSA severity as well as detail information of respective treatments. Although we have included CPAP usage, it might be significantly underestimated using the claim code system. The NIS database also does not collect data on previous acute exacerbations, admissions, or the exact date of receiving prior CABGs or other procedures, hindering further adjustments. This study also lacks information regarding clinical laboratory parameters, preoperative performance status, medications prescribed, number of hospitalizations, and follow-up data, precluding investigation of long-term outcomes such as quality of life.

## Conclusions

In US adults who undergo CABG, compared to COPD alone, patients with OSA-COPD overlap face increased risks of non-routine discharge, respiratory failure/prolonged mechanical ventilation, and AKI after surgery. Conversely, patients with OSA-COPD overlap exhibit a reduced risk of in-hospital mortality compared to those with COPD only. No increased risk of AF was noted. The findings indicate that in cases where OSA and COPD coexist, special attention and heightened vigilance are warranted when considering CABG.

### Supplementary Information

**Supplementary Material 1.**

## Data Availability

The datasets used and/or analysed during the current study are available from the corresponding author on reasonable request.

## Abbreviations

OSA: Obstructive sleep apnea
COPD: Chronic obstructive pulmonary disease
CABG: Coronary artery bypass grafting
AKI: Acute kidney injury
AF: Postoperative atrial fibrillation
VTEs: Venous thromboembolic events
