# Supplementary material for: Outcomes of coronary artery bypass grafting (CABG) in patients with OSA-COPD overlap syndrome versus COPD alone: an analysis of US Nationwide Inpatient Sample
Source: BMC Pulm Med. 2024 Apr 8;24:171. doi: 10.1186/s12890-024-02994-y (PMC11003138; doi:10.1186/s12890-024-02994-y)
Supplement: Supplementary file 1 — Supplementary Material 1. [file 12890_2024_2994_MOESM1_ESM.docx]

| Supplemental Table S1. ICD codes used in the study | | |
| --- | --- | --- |
| Diagnosis | **ICD-9 code** | **ICD-10 code** |
| CABG | **CM**: 36.10-36.17, 36.19, 36.2, 36.31, 36.32, 36.39 | **CM**: 0210, 0211, 0212, 0213, 0214, 0215, 0216, 0217, 021K, 021L, 021P, 021Q, 021R, 021V, 021W, 021X |
| COPD | **CM**: 491, 492, 496 | **CM**: J41-J44 |
| Combine valve surgery | **PCS**: 35.10-35.14, 35.20-35.28 | **PCS**: 024F, 024G, 024J, 025F0ZZ, 025G0ZZ, 025H0ZZ, 025J0ZZ, 02BJ0, 02BH0, 02BG0, 02BF0, 02Q50ZZ, 02QM0ZZ, 02QF0ZZ, 02QG0ZZ, 02QH0ZZ, 02QJ0ZZ |
| Asthma | **CM**: 493 | **CM**: J95 |
| OSA-COPD | **CM**: 327.232 | **CM**: G47.33 |
| Bleeding/ need for  transfusion | **CM**: 285.1, 998.1, 459.0, V58.2, 998.2, 998.51  **PCS**: 39.98, 99.0 | **CM**: I97.4, I97.6, D62, R58, E87.71, J95.84, E83.111, T80.3, T80.4, T80.A, T80.5, T80.6  **PCS**: 30233H, 30233J, 30233K, 30233L, 30233M, 30233N, 30233P, 30233Q, 30233R, 30233S, 30233T, 30233U, 30233V, 30233W, 30233X, 30233Y, 0W38*, 0W3C*, 0W3D* |
| Postoperative shock | **CM**: 998.0, 785.51, 785.50, 785.59, 780.2, 429.4 | **CM**: T81.1, R57.0, R57.1, R57.9, R55, T78.2, I97.1, R65.1 |
| VTE | **CM**: 415, 451-453, 671, 673, 997.2 | **CM**: I260, I269, I801-803, I808, I809, I820-I823, I828, I829, O082, O223, O871, O882, I81, I82 |
| Pneumonia | **CM**: 486, 481, 482.8, 482.3 | **CM**: A48.1, J12-J18 |
| Infection/ Sepsis | **CM**: 995.9, 996.64, 038, 999.3, 790.7, 041, 785.52, 995.0 | **CM**: R78.81, A41, R65.2, T81.4, T80.2, A42.7, A22.7, B37.7, A26.7, A28.2, A54.86, B00.7, A32.7, A24.1, A39.2, A20.7, A21.7, A48.3, A39-A41, T82.6, T82.7, T85.7, T83.5, T83.6, T84.5, T84.6, T84.7 |
| Respiratory failure/  prolonged mechanical ventilation | **CM**:518.5, 518.81-518.84  **PCS**: 96.7 | **CM**: J95.2-J95.8, J96.00, J96.90, J80, J81.0  **PCS**: 5A1955Z |
| AKI | **CM**: 584  DXCCS: 157 | **CM**: N17  DXCCSR_GEN002 >0 |
| Postoperative atrial  fibrillation | **CM**: 997.1 | **CM**: I97.89 |
| Smoking | **CM**: 305.1, V15.82, 989.84 | **CM**: Z71.6, Z72.0, Z86.43, Z87.891, F17, O99.33, T65.2 |
| Congestive heart  failure | **CM**: 398.91, 402.01, 402.11, 402.91, 404.01, 404.03, 404.11, 404.13, 404.91, 404.93, 425.4-425.9, 428 | **CM**: I09.9, I11.0, I13.0, I13.2, I25.5, I42.0, I42.5-I42.9, I43, I50, P29.0 |
| Valvular heart disease | DXCCS: 96 | DXCCSR_CIR001 >0, DXCCSR_CIR003 >0 |
| Chronic kidney disease | **CM**: 403.01, 403.11, 403.91, 404.02, 404.03, 404.12, 404.13, 404.92, 404.93, 582, 583.0-583.7, 585, 586, 588.0, V42.0, V45.1, V56 | **CM**: I12.0, I13.1, N03.2-N03.7, N05.2-N05.7, N18, N19, N25.0, Z49.0-Z49.2, Z94.0, Z99.2 |
| Peripheral vascular  disease | **CM**: 093.0, 437.3, 440, 441, 443.1-443.9, 447.1, 557.1, 557.9, V43.4 | **CM**: I70, I71, I73.1, I73.8, I73.9, I77.1, I79.0, I79.2, K55.1, K55.8, K55.9, Z95.8, Z95.9 |
| Obesity | **CM**: 278.00, 278.01, V85.3, V85.4 | **CM**: E66.01, E66.09, E66.1, E66.2, E66.8, E66.9, Z68.3, Z68.4 |
| Chronic pulmonary  disease | **CM**: 416.8, 416.9, 490-505, 506.4, 508.1, 508.8 | **CM**: I27.8, I27.9, J40-J47, J60-J67, J68.4, J70.1, J70.3 |
| History of MI | **CM**: 412 | **CM**: I25.2 |
| CPAP | **PCS**: 93.9 | **PCS**: 5A09357, 5A09457, 5A09557 |
| Prior PCI | **CM**: V45.82 | **CM**: Z98.61 |
| Prior valvular surgery | **CM**: V42.2, V43.3 | **CM**: Z95.2, Z95.3 |
| Prior CABG | **CM**: V45.81 | **CM**: Z95.1 |
| History of AF | **CM**: 427.3 | **CM**: I48 |
| Diabetes | **CM**: 250 | **CM**: E10-E14 |
| Abbreviation: CABG, coronary artery bypass surgery; OSA, obstructive sleep apnea; COPD, chronic obstructive pulmonary disease; VTE, venous thromboembolism; AKI, acute kidney injury; MI, myocardial infarction; CPAP, continuous positive airway pressure; PCI, percutaneous coronary intervention; AF, atrial fibrillation. | | |

| Supplementary Table S2. Characteristics and outcomes of the study population before PSM | | | | |
| --- | --- | --- | --- | --- |
| Characteristics | Total  (N = 32,190) | OSA-COPD overlap  (n = 3,640) | COPD alone  (n = 28,550) | p |
| **In-hospital mortality** | 572 (1.8) | 40 (1.1) | 532 (1.9) | **<0.001** |
| **LOS, days ^a^** | 8.5 ± 0.04 | 8.5 ± 0.08 | 8.6 ± 0.05 | 0.440 |
| **Non-routine discharge ^a^** | 5320 (21.2) | 511 (24.2) | 4809 (20.9) | **<0.001** |
| **Total hospital costs, per 1000 dollars** | 142.5 ± 1.4 | 155.2 ± 1.9 | 140.8 ± 1.4 | **<0.001** |
| **Postoperative complications** | 21789 (67.8) | 2629 (72.3) | 19160 (67.2) | **<0.001** |
| Bleeding | 15944 (49.7) | 1817 (50.1) | 14127 (49.7) | 0.658 |
| Postoperative shock | 1526 (4.8) | 190 (5.3) | 1336 (4.7) | 0.141 |
| VTE | 597 (1.8) | 74 (2.0) | 523 (1.8) | 0.391 |
| Pneumonia | 1873 (5.8) | 230 (6.3) | 1643 (5.8) | 0.169 |
| Infection/sepsis | 2063 (6.4) | 209 (5.7) | 1854 (6.5) | 0.067 |
| Respiratory failure / prolonged  mechanical ventilation | 6979 (21.6) | 984 (27.0) | 5995 (20.9) | **<0.001** |
| AKI | 4279 (13.4) | 669 (18.4) | 3610 (12.7) | **<0.001** |
| Postoperative AF | 3730 (11.5) | 421 (11.5) | 3309 (11.6) | 0.912 |
| **Age, years** | 66.1 ± 0.1 | 65.1 ± 0.1 | 66.2 ± 0.1 | **<0.001** |
| 18-49 | 1417 (4.4) | 168 (4.6) | 1249 (4.4) | **<0.001** |
| 50-59 | 6365 (19.8) | 800 (22.0) | 5565 (19.5) |  |
| 60-69 | 12434 (38.7) | 1476 (40.5) | 10958 (38.4) |  |
| 70-79 | 9873 (30.6) | 1026 (28.2) | 8847 (31.0) |  |
| 80+ | 2101 (6.5) | 170 (4.7) | 1931 (6.8) |  |
| **Sex** |  |  |  | **<0.001** |
| Male | 23921 (74.3) | 2917 (80.1) | 21004 (73.6) |  |
| Female | 8269 (25.7) | 723 (19.9) | 7546 (26.4) |  |
| **Race** |  |  |  | **0.007** |
| White | 27846 (86.5) | 3201 (87.9) | 24645 (86.4) |  |
| Black | 1621 (5.1) | 190 (5.2) | 1431 (5.0) |  |
| Hispanic | 1488 (4.6) | 146 (4.0) | 1342 (4.7) |  |
| Others | 1235 (3.8) | 103 (2.8) | 1132 (3.9) |  |
| **Insurance status** |  |  |  | **<0.001** |
| Medicare/Medicaid | 21602 (67.3) | 2361 (65.0) | 19241 (67.6) |  |
| Private including HMO | 8822 (27.4) | 1115 (30.6) | 7707 (26.9) |  |
| Self-pay/no-charge/other | 1719 (5.3) | 159 (4.3) | 1560 (5.5) |  |
| Missing | 47 | 5 | 42 |  |
| **Household income** |  |  |  | **0.003** |
| Quartile1 | 10494 (33.3) | 1126 (31.6) | 9368 (33.6) |  |
| Quartile2 | 9349 (29.7) | 1023 (28.7) | 8326 (29.8) |  |
| Quartile3 | 7206 (22.9) | 854 (23.9) | 6352 (22.7) |  |
| Quartile4 | 4460 (14.1) | 567 (15.9) | 3893 (13.9) |  |
| Missing | 681 | 70 | 611 |  |
| **Smoking** |  |  |  | 0.708 |
| No | 10877 (33.5) | 1240 (33.8) | 9637 (33.5) |  |
| Yes | 21313 (66.5) | 2400 (66.2) | 18913 (66.5) |  |
| **Study year** |  |  |  | **<0.001** |
| 2005-2009 | 12127 (36.5) | 685 (18.2) | 11442 (38.9) |  |
| 2010-2015 | 12945 (40.7) | 1703 (46.9) | 11242 (39.9) |  |
| 2016-2018 | 7118 (22.8) | 1252 (34.9) | 5866 (21.2) |  |
| **Hospital bed number** |  |  |  | **0.003** |
| Small | 2932 (8.8) | 347 (9.4) | 2585 (8.8) |  |
| Medium | 7793 (24.5) | 970 (26.9) | 6823 (24.2) |  |
| Large | 21304 (66.7) | 2304 (63.8) | 19000 (67.1) |  |
| Missing | 161 | 19 | 142 |  |
| **Hospital location/**  **teaching status** |  |  |  | **<0.001** |
| Rural | 1592 (4.9) | 136 (3.8) | 1456 (5.1) |  |
| Urban nonteaching | 11814 (36.5) | 1130 (31.0) | 10684 (37.2) |  |
| Urban teaching | 18623 (58.6) | 2355 (65.2) | 16268 (57.7) |  |
| Missing | 161 | 19 | 142 |  |
| **Comorbidity** |  |  |  |  |
| Congestive heart failure | 8891 (27.7) | 1119 (30.9) | 7772 (27.3) | **<0.001** |
| Valvular heart disease | 3051 (9.5) | 320 (8.8) | 2731 (9.6) | 0.119 |
| Chronic kidney disease | 4789 (14.9) | 760 (21.0) | 4029 (14.2) | **<0.001** |
| Peripheral vascular disease | 8414 (26.2) | 815 (22.4) | 7599 (26.6) | **<0.001** |
| Obesity | 6976 (21.8) | 1938 (53.3) | 5038 (17.8) | **<0.001** |
| History of MI | 6510 (20.3) | 779 (21.5) | 5731 (20.2) | 0.059 |
| History of AF | 10355 (32.3) | 1370 (37.8) | 8985 (31.6) | **<0.001** |
| Diabetes | 12834 (40.0) | 2048 (56.3) | 10786 (37.9) | **<0.001** |
| CPAP | 1401 (4.4) | 373 (10.3) | 1028 (3.6) | **<0.001** |
| Prior PCI | 3642 (11.3) | 444 (12.2) | 3198 (11.2) | 0.065 |
| Prior valvular surgery | 89 (0.3) | 14 (0.4) | 75 (0.3) | 0.244 |
| Prior CABG | 583 (1.8) | 77 (2.1) | 506 (1.8) | 0.146 |
| **ACCI** |  |  |  | **<0.001** |
| 0 | 306 (0.9) | 26 (0.7) | 280 (1.0) |  |
| 1 | 1852 (5.7) | 192 (5.2) | 1660 (5.8) |  |
| 2 | 4705 (14.6) | 517 (14.1) | 4188 (14.6) |  |
| 3 | 7049 (21.9) | 702 (19.3) | 6347 (22.2) |  |
| 4 | 6875 (21.4) | 739 (20.3) | 6136 (21.5) |  |
| 5+ | 11403 (35.5) | 1464 (40.4) | 9939 (34.9) |  |
| Abbreviations: LOS, length of hospital stays; VTE, venous thromboembolism; AKI, acute kidney injury; AF, atrial fibrillation; HMO, Health Maintenance Organization; CKD, chronic kidney disease; MI, myocardial infarction; CPAP, continuous positive airway pressure; PCI, percutaneous coronary intervention; CABG, coronary artery bypass grafting; ACCI, age-adjusted Charlson Comorbidity Index.  Continuous variables are presented as mean ± SE; categorical variables are presented as unweighted counts (weighted percentage).  a Excluding patients who died in the hospital.  p-values < 0.05 are shown in bold. | | | | |

| Supplementary Table S3. Associations between covariates and in-hospital outcomes | | | | |
| --- | --- | --- | --- | --- |
|  | In-hospital mortality | | LOS ^a,^ | |
|  | OR (95% CI) | aOR (95% CI) | Beta (95% CI) | aBeta (95% CI) |
| **OSA-COPD overlap VS. COPD alone** | **0.56 (0.37, 0.85)** | **0.54 (0.36, 0.82)** | -0.03 (-0.25, 0.19) | -0.19 (-0.42, 0.04) |
| **Age, years** |  |  |  |  |
| 18-49 | Ref. |  | Ref. |  |
| 50-59 | NA |  | **0.32 (0.00, 0.64)** |  |
| 60-69 | NA |  | **0.80 (0.49, 1.10)** |  |
| 70-79 | NA |  | **1.94 (1.61, 2.27)** |  |
| 80+ | NA |  | **3.42 (2.86, 3.98)** |  |
| **Sex** |  |  |  |  |
| Male | 0.99 (0.71, 1.37) |  | **-0.30 (-0.52, -0.09)** | -0.19 (-0.40, 0.03) |
| Female | Ref. |  | Ref. | Ref. |
| **Race** |  |  |  |  |
| White | Ref. |  | Ref. | Ref. |
| Black | 1.18 (0.66, 2.13) |  | **0.86 (0.29, 1.42)** | **0.77 (0.21, 1.34)** |
| Hispanic | 1.16 (0.61, 2.19) |  | **0.68 (0.20, 1.16)** | **0.55 (0.06, 1.05)** |
| Others | 1.17 (0.60, 2.30) |  | 0.05 (-0.37, 0.47) | -0.05 (-0.47, 0.37) |
| **Insurance status** |  |  |  |  |
| Medicare/Medicaid | Ref. | Ref. | Ref. | Ref. |
| Private including HMO | **0.51 (0.35, 0.74)** | **0.55 (0.38, 0.79)** | **-1.14 (-1.34, -0.93)** | **-1.00 (-1.21, -0.79)** |
| Self-pay/no-charge/other | 0.58 (0.27, 1.25) | 0.63 (0.29, 1.36) | **-0.47 (-0.87, -0.07)** | -0.24 (-0.65, 0.17) |
| **Household income** |  |  |  |  |
| Quartile1 | 1.32 (0.82, 2.12) |  | -0.23 (-0.57, 0.11) | -0.23 (-0.57, 0.11) |
| Quartile2 | 1.23 (0.75, 2.03) |  | -0.22 (-0.56, 0.13) | -0.16 (-0.50, 0.18) |
| Quartile3 | 0.98 (0.58, 1.66) |  | -0.28 (-0.64, 0.08) | -0.25 (-0.60, 0.11) |
| Quartile4 | Ref. |  | Ref. | Ref. |
| **Smoking** |  |  |  |  |
| No | Ref. | Ref. | Ref. | Ref. |
| Yes | **0.47 (0.35, 0.63)** | **0.48 (0.36, 0.64)** | **-1.88 (-2.13, -1.63)** | **-1.77 (-2.03, -1.52)** |
| **Study year** |  |  |  |  |
| 2005-2009 | Ref. |  |  |  |
| 2010-2015 | **1.63 (1.08, 2.46)** |  | -0.29 (-0.56, -0.02) |  |
| 2016-2018 | **1.99 (1.32, 3.00)** |  | -0.14 (-0.43, 0.15) |  |
| **Hospital bed number** |  |  |  |  |
| Small | **1.60 (1.03, 2.48)** | **1.62 (1.05, 2.52)** | **-0.39 (-0.72, -0.06)** | **-0.38 (-0.71, -0.04)** |
| Medium | **1.40 (1.01, 1.94)** | **1.47 (1.05, 2.04)** | -0.21 (-0.45, 0.03) | -0.20 (-0.45, 0.05) |
| Large | Ref. | Ref. | Ref. | Ref. |
| **Hospital location/ teaching status** |  |  |  |  |
| Rural | 1.55 (0.93, 2.60) |  | -0.74 (-1.09, -0.40) | -0.80 (-1.18, -0.42) |
| Urban nonteaching | 1.09 (0.80, 1.49) |  | -0.14 (-0.36, 0.08) | -0.16 (-0.38, 0.07) |
| Urban teaching | Ref. |  | Ref. | Ref. |
| **Comorbidity** |  |  |  |  |
| Congestive heart failure | **2.19 (1.66, 2.89)** |  | **2.31 (2.07, 2.56)** |  |
| Valvular heart disease | **1.79 (1.18, 2.73)** |  | **0.69 (0.33, 1.05)** |  |
| Chronic kidney disease | **2.60 (1.94, 3.49)** |  | **2.15 (1.83, 2.47)** |  |
| Peripheral vascular disease | **1.57 (1.17, 2.09)** |  | 0.11 (-0.10, 0.32) |  |
| Obesity | **0.56 (0.40, 0.80)** |  | 0.13 (-0.09, 0.35) |  |
| History of MI | 0.74 (0.51, 1.06) |  | **-0.84 (-1.05, -0.63)** |  |
| History of AF | **1.66 (1.25, 2.22)** |  | **2.15 (1.93, 2.38)** |  |
| Diabetes | **0.70 (0.53, 0.93)** |  | 0.00 (-0.19, 0.19) |  |
| CPAP | 1.51 (0.87, 2.63) |  | **1.63 (1.16, 2.10)** | **1.60 (1.12, 2.07)** |
| Prior PCI | **0.50 (0.27, 0.91)** | 0.56 (0.31, 1.02) | **-0.79 (-1.03, -0.55)** | **-0.61 (-0.86, -0.37)** |
| Prior valvular surgery | NA |  | 0.44 (-0.79, 1.68) | 0.11 (-1.15, 1.37) |
| Prior CABG | 1.35 (0.55, 3.31) |  | -0.01 (-0.91, 0.89) | 0.04 (-0.88, 0.96) |
| **ACCI** |  |  |  |  |
| 0 | Ref. |  | Ref. |  |
| 1 | NA |  | **0.55 (0.08, 1.03)** |  |
| 2 | NA |  | **0.90 (0.46, 1.33)** |  |
| 3 | NA |  | **1.26 (0.84, 1.68)** |  |
| 4 | NA |  | **1.98 (1.56, 2.40)** |  |
| 5+ | NA |  | **3.29 (2.88, 3.70)** |  |
| Abbreviations: LOS, length of hospital stays; AF, atrial fibrillation; HMO, Health Maintenance Organization; CKD, chronic kidney disease; MI, myocardial infarction; CPAP, continuous positive airway pressure; PCI, percutaneous coronary intervention; CABG, coronary artery bypass grafting; CCI, Charlson Comorbidity Index. OR, odds ratio; aOR, adjusted odds ratio; CI, confidence interval.  ^a^ Excluding patients who died in the hospital.  p-value < 0.05 are showed in bold. | | | | |

| Supplementary Table S3. Associations between covariates and in-hospital outcomes (cont.) | | | | |
| --- | --- | --- | --- | --- |
|  | Non-routine discharge ^a^ | | Postoperative complications, any | |
|  | OR (95% CI) | aOR (95% CI) | OR (95% CI) | aOR (95% CI) |
| **OSA-COPD overlap VS. COPD alone** | **1.21 (1.09, 1.35)** | **1.17 (1.05, 1.31)** | **1.18 (1.07, 1.31)** | **1.13 (1.02, 1.25)** |
| **Age, years** |  |  |  |  |
| 18-49 | Ref. |  | Ref. |  |
| 50-59 | **1.72 (1.11, 2.65)** |  | 0.96 (0.80, 1.16) |  |
| 60-69 | **4.04 (2.68, 6.11)** |  | 1.13 (0.95, 1.36) |  |
| 70-79 | **10.02 (6.66, 15.10)** |  | **1.48 (1.22, 1.78)** |  |
| 80+ | **26.72 (17.37, 41.11)** |  | **1.89 (1.49, 2.41)** |  |
| **Sex** |  |  |  |  |
| Male | **0.58 (0.53, 0.64)** | **0.61 (0.55, 0.68)** | **0.76 (0.69, 0.83)** | **0.78 (0.71, 0.85)** |
| Female | Ref. | Ref. | Ref. | Ref. |
| **Race** |  |  |  |  |
| White | Ref. |  | Ref. | Ref. |
| Black | 1.18 (0.97, 1.43) |  | **1.42 (1.18, 1.71)** | **1.41 (1.17, 1.71)** |
| Hispanic | 1.04 (0.85, 1.27) |  | **1.43 (1.14, 1.79)** | **1.45 (1.15, 1.82)** |
| Others | 0.94 (0.74, 1.19) |  | **1.31 (1.06, 1.63)** | **1.31 (1.05, 1.63)** |
| **Insurance status** |  |  |  |  |
| Medicare/Medicaid | Ref. | Ref. | Ref. | Ref. |
| Private including HMO | **0.26 (0.23, 0.29)** | **0.27 (0.23, 0.31)** | **0.76 (0.69, 0.82)** | **0.77 (0.71, 0.85)** |
| Self-pay/no-charge/other | **0.22 (0.16, 0.30)** | **0.24 (0.17, 0.32)** | **0.72 (0.60, 0.85)** | **0.77 (0.65, 0.91)** |
| **Household income** |  |  |  |  |
| Quartile1 | **0.84 (0.72, 0.97)** |  | **0.81 (0.71, 0.93)** | **0.80 (0.69, 0.92)** |
| Quartile2 | 0.93 (0.80, 1.08) |  | 0.89 (0.78, 1.02) | 0.91 (0.79, 1.04) |
| Quartile3 | 0.99 (0.85, 1.15) |  | 0.93 (0.80, 1.07) | 0.94 (0.81, 1.08) |
| Quartile4 | Ref. |  | Ref. | Ref. |
| **Smoking** |  |  |  |  |
| No | Ref. | Ref. | Ref. | Ref. |
| Yes | **0.56 (0.52, 0.62)** | **0.61 (0.56, 0.67)** | **0.78 (0.72, 0.85)** | **0.82 (0.75, 0.89)** |
| **Study year** |  |  |  |  |
| 2005-2009 | Ref. |  | Ref. |  |
| 2010-2015 | **1.39 (1.22, 1.58)** |  | **1.42 (1.26, 1.60)** |  |
| 2016-2018 | **1.78 (1.57, 2.02)** |  | **1.35 (1.20, 1.52)** |  |
| **Hospital bed number** |  |  |  |  |
| Small | **1.19 (1.03, 1.39)** |  | 0.98 (0.85, 1.14) |  |
| Medium | 0.99 (0.89, 1.11) |  | 0.97 (0.87, 1.08) |  |
| Large | Ref. |  | Ref. |  |
| **Hospital location/ teaching status** |  |  |  |  |
| Rural | **0.71 (0.53, 0.94)** |  | **0.71 (0.56, 0.90)** | 0.80 (0.63, 1.01) |
| Urban nonteaching | 0.97 (0.88, 1.08) |  | **0.86 (0.78, 0.95)** | **0.87 (0.78, 0.96)** |
| Urban teaching | Ref. |  | Ref. | Ref. |
| **Comorbidity** |  |  |  |  |
| Congestive heart failure | **1.90 (1.73, 2.08)** |  | **1.60 (1.46, 1.75)** |  |
| Valvular heart disease | 1.15 (0.99, 1.33) |  | **1.30 (1.13, 1.50)** |  |
| Chronic kidney disease | **2.52 (2.27, 2.80)** |  | **2.83 (2.49, 3.22)** |  |
| Peripheral vascular disease | **1.24 (1.13, 1.37)** |  | **1.22 (1.12, 1.34)** |  |
| Obesity | **1.32 (1.21, 1.45)** |  | 0.94 (0.86, 1.02) |  |
| History of MI | 0.91 (0.81, 1.01) |  | **0.80 (0.73, 0.87)** |  |
| History of AF | **1.99 (1.82, 2.18)** |  | **2.10 (1.92, 2.30)** |  |
| Diabetes | **1.34 (1.23, 1.46)** |  | **1.12 (1.04, 1.20)** |  |
| CPAP | **1.97 (1.65, 2.37)** | **1.86 (1.53, 2.27)** | **2.12 (1.68, 2.67)** | **2.05 (1.62, 2.60)** |
| Prior PCI | **0.71 (0.61, 0.83)** | **0.75 (0.64, 0.89)** | **0.87 (0.77, 0.98)** | 0.90 (0.79, 1.02) |
| Prior valvular surgery | 0.63 (0.24, 1.62) |  | 1.20 (0.58, 2.48) |  |
| Prior CABG | 0.73 (0.51, 1.04) |  | 0.99 (0.75, 1.30) |  |
| **ACCI** |  |  |  |  |
| 0 | Ref. |  | Ref. |  |
| 1 | NA |  | 0.97 (0.65, 1.46) |  |
| 2 | NA |  | 1.01 (0.69, 1.48) |  |
| 3 | NA |  | 1.23 (0.84, 1.80) |  |
| 4 | NA |  | **1.48 (1.01, 2.18)** |  |
| 5+ | NA |  | **2.27 (1.55, 3.34)** |  |
| Abbreviations: AF, atrial fibrillation; HMO, Health Maintenance Organization; CKD, chronic kidney disease; MI, myocardial infarction; CPAP, continuous positive airway pressure; PCI, percutaneous coronary intervention; CABG, coronary artery bypass grafting; CCI, Charlson Comorbidity Index. OR, odds ratio; aOR, adjusted odds ratio; CI, confidence interval.  ^a^ Excluding patients who died in the hospital.  p-value < 0.05 are showed in bold. | | | | |

| Supplementary Table S3. Associations between covariates and in-hospital outcomes (cont.) | | | | |
| --- | --- | --- | --- | --- |
|  | Pneumonia | | Respiratory failure / prolonged mechanical ventilation | |
|  | OR (95% CI) | aOR (95% CI) | OR (95% CI) | aOR (95% CI) |
| **OSA-COPD overlap VS. COPD alone** | 1.04 (0.87, 1.25) | 0.96 (0.79, 1.17) | **1.37 (1.24, 1.51)** | **1.26 (1.13, 1.40)** |
| **Age, years** |  |  |  |  |
| 18-49 | Ref. |  | Ref. |  |
| 50-59 | 0.75 (0.53, 1.06) |  | 0.87 (0.70, 1.07) |  |
| 60-69 | 0.67 (0.49, 0.94) |  | 0.85 (0.69, 1.04) |  |
| 70-79 | 0.75 (0.54, 1.04) |  | 0.92 (0.75, 1.14) |  |
| 80+ | 1.15 (0.77, 1.71) |  | 0.94 (0.73, 1.22) |  |
| **Sex** |  |  |  |  |
| Male | **1.27 (1.05, 1.54)** | **1.38 (1.14, 1.67)** | **0.84 (0.76, 0.92)** | **0.86 (0.78, 0.95)** |
| Female | Ref. | Ref. | Ref. | Ref. |
| **Race** |  |  |  |  |
| White | Ref. | Ref. | Ref. | Ref. |
| Black | 1.03 (0.74, 1.43) | 1.03 (0.74, 1.44) | 1.19 (0.99, 1.43) | 1.15 (0.95, 1.38) |
| Hispanic | 1.23 (0.87, 1.75) | 1.17 (0.82, 1.68) | **1.62 (1.34, 1.95)** | **1.59 (1.31, 1.92)** |
| Others | 1.14 (0.78, 1.68) | 1.05 (0.70, 1.57) | 1.23 (0.99, 1.53) | 1.24 (0.99, 1.56) |
| **Insurance status** |  |  |  |  |
| Medicare/Medicaid | Ref. | Ref. | Ref. | Ref. |
| Private including HMO | **0.73 (0.61, 0.88)** | **0.75 (0.62, 0.90)** | 0.94 (0.85, 1.03) | 0.95 (0.86, 1.05) |
| Self-pay/no-charge/other | 0.91 (0.65, 1.28) | 0.94 (0.66, 1.35) | 0.96 (0.79, 1.17) | 1.04 (0.86, 1.27) |
| **Household income** |  |  |  |  |
| Quartile1 | 1.15 (0.91, 1.45) | 1.19 (0.93, 1.52) | 0.94 (0.82, 1.09) | 1.00 (0.86, 1.16) |
| Quartile2 | 1.00 (0.78, 1.27) | 1.02 (0.79, 1.30) | 0.99 (0.86, 1.15) | 1.05 (0.90, 1.22) |
| Quartile3 | 0.90 (0.70, 1.17) | 0.92 (0.70, 1.19) | 0.98 (0.84, 1.15) | 1.00 (0.85, 1.17) |
| Quartile4 | Ref. |  | Ref. | Ref. |
| **Smoking** |  |  |  |  |
| No | Ref. | Ref. | Ref. | Ref. |
| Yes | **0.63 (0.55, 0.74)** | **0.66 (0.57, 0.77)** | **0.74 (0.68, 0.81)** | **0.77 (0.70, 0.85)** |
| **Study year** |  |  |  |  |
| 2005-2009 | Ref. |  | Ref. |  |
| 2010-2015 | 1.08 (0.89, 1.32) |  | 1.13 (0.98, 1.29) |  |
| 2016-2018 | 1.15 (0.94, 1.41) |  | **0.69 (0.60, 0.80)** |  |
| **Hospital bed number** |  |  |  |  |
| Small | 1.11 (0.86, 1.43) |  | 0.91 (0.77, 1.07) |  |
| Medium | 1.12 (0.94, 1.34) |  | 0.89 (0.78, 1.00) |  |
| Large | Ref. |  | Ref. |  |
| **Hospital location/ teaching status** |  |  |  |  |
| Rural | 1.07 (0.76, 1.51) | 0.95 (0.65, 1.38) | **0.64 (0.47, 0.87)** | **0.66 (0.49, 0.90)** |
| Urban nonteaching | **1.19 (1.01, 1.39)** | **1.19 (1.01, 1.41)** | **1.14 (1.02, 1.27)** | **1.13 (1.01, 1.27)** |
| Urban teaching | Ref. | Ref. | Ref. | Ref. |
| **Comorbidity** |  |  |  |  |
| Congestive heart failure | **1.85 (1.59, 2.15)** |  | **1.43 (1.30, 1.58)** |  |
| Valvular heart disease | 1.11 (0.86, 1.43) |  | 1.09 (0.94, 1.25) |  |
| Chronic kidney disease | **1.56 (1.31, 1.86)** |  | **1.32 (1.18, 1.48)** |  |
| Peripheral vascular disease | 1.18 (1.00, 1.39) |  | 1.02 (0.92, 1.12) |  |
| Obesity | 0.94 (0.79, 1.10) |  | 1.07 (0.97, 1.18) |  |
| History of MI | **0.63 (0.51, 0.78)** |  | **0.76 (0.69, 0.85)** |  |
| History of AF | **1.56 (1.34, 1.81)** |  | **1.35 (1.23, 1.47)** |  |
| Diabetes | 0.95 (0.82, 1.11) |  | 1.04 (0.95, 1.14) |  |
| CPAP | **2.49 (1.94, 3.21)** | **2.65 (2.03, 3.46)** | **2.84 (2.38, 3.38)** | **2.73 (2.28, 3.28)** |
| Prior PCI | **0.59 (0.44, 0.80)** | **0.62 (0.46, 0.84)** | 0.99 (0.86, 1.14) | 0.98 (0.85, 1.13) |
| Prior valvular surgery | NA |  | 0.41 (0.14, 1.15) |  |
| Prior CABG | 0.79 (0.44, 1.42) |  | 0.94 (0.69, 1.29) |  |
| **ACCI** |  |  |  |  |
| 0 | Ref. |  | Ref. |  |
| 1 | 1.07 (0.52, 2.21) |  | 0.83 (0.53, 1.31) |  |
| 2 | 0.57 (0.29, 1.14) |  | 0.69 (0.44, 1.07) |  |
| 3 | 0.67 (0.34, 1.33) |  | 0.78 (0.51, 1.21) |  |
| 4 | 0.81 (0.41, 1.59) |  | 0.84 (0.55, 1.31) |  |
| 5+ | 1.10 (0.57, 2.14) |  | 0.95 (0.62, 1.46) |  |
| Abbreviations: AF, atrial fibrillation; HMO, Health Maintenance Organization; CKD, chronic kidney disease; MI, myocardial infarction; CPAP, continuous positive airway pressure; PCI, percutaneous coronary intervention; CABG, coronary artery bypass grafting; CCI, Charlson Comorbidity Index. OR, odds ratio; aOR, adjusted odds ratio; CI, confidence interval.  p-value < 0.05 are showed in bold. | | | | |

| Supplementary Table S3. Associations between covariates and in-hospital outcomes (cont.) | | | | |
| --- | --- | --- | --- | --- |
|  | AKI | | Postoperative AF | |
|  | OR (95% CI) | aOR (95% CI) | OR (95% CI) | aOR (95% CI) |
| **OSA-COPD overlap VS. COPD alone** | 1.04 (0.87, 1.25) | **1.16 (1.03, 1.32)** | **1.37 (1.24, 1.51)** | 1.03 (0.89, 1.18) |
| **Age, years** |  |  |  |  |
| 18-49 | Ref. |  | Ref. |  |
| 50-59 | 1.15 (0.83, 1.59) |  | 1.29 (0.90, 1.86) |  |
| 60-69 | **1.65 (1.21, 2.25)** |  | **1.56 (1.10, 2.20)** |  |
| 70-79 | **2.57 (1.89, 3.49)** |  | **1.92 (1.36, 2.73)** |  |
| 80+ | **4.33 (3.10, 6.06)** |  | **2.46 (1.66, 3.64)** |  |
| **Sex** |  |  |  |  |
| Male | **1.27 (1.12, 1.45)** | **1.36 (1.20, 1.55)** | 0.93 (0.81, 1.05) | 0.94 (0.82, 1.07) |
| Female | Ref. | Ref. | Ref. | Ref. |
| **Race** |  |  |  |  |
| White | Ref. | Ref. | Ref. | Ref. |
| Black | **1.43 (1.16, 1.77)** | **1.47 (1.18, 1.82)** | 0.85 (0.65, 1.11) | 0.87 (0.67, 1.14) |
| Hispanic | **1.36 (1.07, 1.72)** | 1.28 (0.99, 1.65) | 0.77 (0.56, 1.04) | 0.77 (0.56, 1.06) |
| Others | 1.08 (0.83, 1.40) | 1.03 (0.78, 1.36) | 0.77 (0.55, 1.06) | 0.73 (0.52, 1.03) |
| **Insurance status** |  |  |  |  |
| Medicare/Medicaid | Ref. | Ref. | Ref. |  |
| Private including HMO | **0.55 (0.48, 0.63)** | **0.56 (0.49, 0.64)** | **0.81 (0.71, 0.93)** | 0.85 (0.74, 0.97) |
| Self-pay/no-charge/other | **0.68 (0.54, 0.87)** | **0.76 (0.59, 0.97)** | 0.78 (0.60, 1.03) | 0.85 (0.64, 1.12) |
| **Household income** |  |  |  |  |
| Quartile1 | 0.95 (0.80, 1.13) | 0.99 (0.83, 1.18) | 0.87 (0.72, 1.05) | 0.85 (0.70, 1.03) |
| Quartile2 | 1.01 (0.85, 1.20) | 1.05 (0.89, 1.25) | 0.83 (0.69, 1.01) | 0.83 (0.68, 1.01) |
| Quartile3 | 1.05 (0.88, 1.25) | 1.08 (0.90, 1.29) | 0.91 (0.75, 1.12) | 0.91 (0.74, 1.12) |
| Quartile4 | Ref. | Ref. | Ref. | Ref. |
| **Smoking** |  |  |  |  |
| No | Ref. | Ref. | Ref. | Ref. |
| Yes | **0.54 (0.49, 0.60)** | **0.57 (0.51, 0.63)** | **0.60 (0.53, 0.67)** | **0.61 (0.54, 0.68)** |
| **Study year** |  |  |  |  |
| 2005-2009 |  |  |  |  |
| 2010-2015 | **1.67 (1.44, 1.93)** |  | 0.99 (0.86, 1.15) |  |
| 2016-2018 | **2.43 (2.10, 2.81)** |  | **0.42 (0.35, 0.50)** |  |
| **Hospital bed number** |  |  |  |  |
| Small | 1.08 (0.90, 1.30) |  | **0.65 (0.52, 0.81)** |  |
| Medium | 0.98 (0.86, 1.11) |  | 0.96 (0.84, 1.11) |  |
| Large | Ref. |  | Ref. |  |
| **Hospital location/ teaching status** |  |  |  |  |
| Rural | **0.47 (0.35, 0.64)** | **0.48 (0.35, 0.65)** | **1.38 (1.06, 1.80)** | **1.48 (1.12, 1.95)** |
| Urban nonteaching | **0.82 (0.73, 0.92)** | **0.84 (0.74, 0.95)** | **1.14 (1.00, 1.31)** | 1.14 (1.00, 1.30) |
| Urban teaching | Ref. | Ref. | Ref. |  |
| **Comorbidity** |  |  |  |  |
| Congestive heart failure | **2.46 (2.21, 2.73)** |  | 1.12 (0.99, 1.27) |  |
| Valvular heart disease | 1.00 (0.84, 1.19) |  | **1.24 (1.02, 1.50)** |  |
| Chronic kidney disease | **10.03 (8.95, 11.25)** |  | 1.14 (0.98, 1.32) |  |
| Peripheral vascular disease | **1.35 (1.21, 1.51)** |  | 1.05 (0.92, 1.20) |  |
| Obesity | 0.97 (0.87, 1.08) |  | 1.05 (0.92, 1.19) |  |
| History of MI | **0.82 (0.72, 0.93)** |  | **0.71 (0.61, 0.82)** |  |
| History of AF | **1.86 (1.68, 2.07)** |  | **7.59 (6.64, 8.68)** |  |
| Diabetes | **1.47 (1.32, 1.62)** |  | **0.80 (0.72, 0.90)** |  |
| CPAP | **1.56 (1.27, 1.91)** | **1.49 (1.20, 1.86)** | **1.47 (1.14, 1.90)** | **1.38 (1.05, 1.81)** |
| Prior PCI | **0.59 (0.49, 0.72)** | **0.62 (0.51, 0.77)** | **0.79 (0.65, 0.97)** | **0.81 (0.66, 1.00)** |
| Prior valvular surgery | 1.18 (0.49, 2.82) |  | 0.51 (0.12, 2.10) |  |
| Prior CABG | 0.95 (0.66, 1.38) |  | **0.45 (0.25, 0.83)** |  |
| **ACCI** |  |  |  |  |
| 0 | Ref. |  | Ref. |  |
| 1 | 1.24 (0.46, 3.31) |  | 1.43 (0.60, 3.38) |  |
| 2 | 1.45 (0.53, 3.92) |  | 1.90 (0.84, 4.32) |  |
| 3 | 2.42 (0.91, 6.46) |  | 1.99 (0.88, 4.53) |  |
| 4 | **3.64 (1.37, 9.70)** |  | **2.42 (1.06, 5.54)** |  |
| 5+ | **12.70 (4.82, 33.46)** |  | 2.22 (0.98, 5.01) |  |
| Abbreviations: AKI, acute kidney injury; AF, atrial fibrillation; HMO, Health Maintenance Organization; CKD, chronic kidney disease; MI, myocardial infarction; CPAP, continuous positive airway pressure; PCI, percutaneous coronary intervention; CABG, coronary artery bypass grafting; CCI, Charlson Comorbidity Index. OR, odds ratio; aOR, adjusted odds ratio; CI, confidence interval.  p-value < 0.05 are showed in bold. | | | | |
